# Evaluation of the Pathogenicity and the Escape from Vaccine Protection of a New Antigenic Variant Derived from the European Human-Like Reassortant Swine H1N2 Influenza Virus

**DOI:** 10.3390/v12101155

**Published:** 2020-10-12

**Authors:** Céline Deblanc, Stéphane Quéguiner, Stéphane Gorin, Amélie Chastagner, Séverine Hervé, Frédéric Paboeuf, Gaëlle Simon

**Affiliations:** 1 Swine Virology Immunology Unit, Ploufragan-Plouzané-Niort Laboratory, French Agency for Food, Environmental and Occupational Health and Safety (ANSES), 22440 Ploufragan, France; stephane.queguiner@anses.fr (S.Q.); stephane.gorin@anses.fr (S.G.); amelie.chastagner@anses.fr (A.C.); severine.herve@anses.fr (S.H.); gaelle.simon@anses.fr (G.S.); 2 SPF Pig Production and Experimentation, Ploufragan-Plouzané-Niort Laboratory, French Agency for Food, Environmental and Occupational Health and Safety (ANSES), 22440 Ploufragan, France; frederic.paboeuf@anses.fr

**Keywords:** swine influenza virus, H1N2, variant, post-infectious immune response, pathogenesis, vaccine

## Abstract

The surveillance of swine influenza A viruses in France revealed the emergence of an antigenic variant following deletions and mutations that are fixed in the HA-encoding gene of the European human-like reassortant swine H1N2 lineage. In this study, we compared the outcomes of the parental (H1_hu_N2) and variant (H1_hu_N2_Δ14–147_) virus infections in experimentally-inoculated piglets. Moreover, we assessed and compared the protection that was conferred by an inactivated vaccine currently licensed in Europe. Three groups of five unvaccinated or vaccinated piglets were inoculated with H1_hu_N2 or H1_hu_N2_Δ14–147_ or mock-inoculated, respectively. In unvaccinated piglets, the variant strain induced greater clinical signs than the parental virus, in relation to a higher inflammatory response that involves TNF-α production and a huge afflux of granulocytes into the lung. However, both infections led to similar levels of virus excretion and adaptive (humoral and cellular) immune responses in blood. The vaccinated animals were clinically protected from both infectious challenges and did not exhibit any inflammatory responses, regardless the inoculated virus. However, whereas vaccination prevented virus shedding in H1_hu_N2-infected animals, it did not completely inhibit the multiplication of the variant strain, since live virus particles were detected in nasal secretions that were taken from H1_hu_N2_Δ14–147_-inoculated vaccinated piglets. This difference in the level of vaccine protection was probably related to the poorer ability of the post-vaccine antibodies to neutralize the variant virus than the parental virus, even though post-vaccine cellular immunity appeared to be equally effective against both viruses. These results suggest that vaccine antigens would potentially need to be updated if this variant becomes established in Europe.

## 1. Introduction

Swine influenza is an acute respiratory infection of pigs that is characterized by fever, loss of appetite, lethargy, dyspnea, nasal discharge, and coughing. Swine influenza A viruses (swIAV), the etiological agents of the disease, are widespread around the world. There are three main swIAV subtypes: H1N1, H1N2, and H3N2, but viruses within subtypes vary, depending on the geographic area. Thus, four distinct genetic lineages are enzootic in pig herds in Europe: the “avian-like swine H1N1” (H1_av_N1), the “pandemic-like swine H1N1” (H1N1pdm), the “human-like reassortant swine H1N2” (H1_hu_N2), and the “human-like reassortant swine H3N2” (H3N2) [1]. Given the impact of swIAV in terms of animal health and public health due to zoonotic potential, monitoring the circulation and evolution of virus strains has been encouraged in many countries after the last influenza pandemic in 2009. In France, swIAV surveillance highlighted the emergence in 2012 of a new antigenic variant of H1N2 subtype, which results from a genetic drift in the H1_hu_N2 virus circulating since the ‘90s (“Scotland/94” lineage; HA clade 1B.1.2.3) [2,3]. The haemagglutinin (HA)-encoding gene of the variant exhibits mutations in several antigenic sites, as well as two successive amino acid deletions at residue positions 146–147 (129–130 when H1 numbering without signal peptide), which are located in the cell receptor binding site (RBS) [2,4]. The proportion of new variant H1_hu_N2_Δ14–147_ strains that were detected over the country among the H1_hu_N2 viruses increased until reaching 50% in 2013–2014 [3]. Interestingly, other swIAV strains exhibiting a H1 glycoprotein with similar amino acid deletions into the RBS at positions 146 and/or 147 have been described elsewhere, whereas belonging to other HA-1B (H1_hu_) clades, e.g., 1B.1.2.2 in Italy [5,6] and 1B.2-1B.1-like in Russia [7], but also to other H1 lineages, e.g., 1A.1.1 (classical swine or H1α3) in North America [8] and 1C.2 (H1_av_) in France [9]. However, the advantage of such modifications for the viruses is still unknown. They may induce changes in cell receptor affinity, viral multiplication, pathogenicity, antigenic properties, or escape to vaccine protection, with potential consequences in virus adaptation to the species and spread into the pig population. To date, no evidence of swine-to-human transmission of such deleted viruses has been reported.

In order to increase knowledge on properties of H1_hu_N2_Δ14–147_ new variant, we conducted an experimental infection assay in pigs (i) to study the clinical, virological, and immunological (innate, cellular and humoral) responses after infection with H1_hu_N2_Δ14–147_ in comparison to those observed after parental H1_hu_N2 virus inoculation and (ii) to compare the protection that is conferred by an anti-H1_hu_N2 inactivated vaccine currently licensed in Europe against these viruses.

## 2. Materials and Methods

### 2.1. Vaccine and Viruses

The vaccine that was used in this study was an adjuvanted inactivated trivalent vaccine (Respiporc Flu^®^3, CEVA, Libourne, France; formerly provided by IDT Biologika GmbH, Dessau-Rosslau, Germany) containing antigens representative of three out of the four most widespread enzootic swIAV lineages circulating in Europe, i.e., “avian-like swine H1N1” (H1_av_N1), “human-like reassortant swine H3N2” (H3N2), and “human-like reassortant swine H1N2” (H1_hu_N2) [1,10]. The H1_hu_N2 antigen included into the vaccine is strain A/Sw/Bakum/1832/2000 (Bakum/00).

The challenge swIAV strains A/Sw/France/Ille et Vilaine-0415/2011 (H1_hu_N2; 415/11) and A/Sw/France/22-130212/2013 (H1_hu_N2_Δ14–147;_ 212/13) were selected among collections of the French National Reference Laboratory for Swine Influenza (ANSES, Ploufragan, France). They were isolated from nasal swabs taken from pigs with acute respiratory disease thanks to swIAV passive surveillance, propagated, and titrated in Madin–Darby canine kidney (MDCK) cells following a standard procedure [11].

Three other H1_hu_N2 strains were used as reference antigens for haemagglutination inhibition (HI) assays: A/Sw/Scotland/410440/94 (Scotland/94), A/Sw/Cotes d’Armor/0214/06 (214/06), and A/Sw/Cotes d’Armor/0113/06 (113/06). The latter is representative of the most prevalent H1_hu_N2 strains that have been circulating in France for a decade [4].

The H1 genes of vaccine antigen Bakum/00, challenge strains 415/11 and 212/13, and other H1_hu_N2 reference strains all belong to clade 1B (“Scotland/94” lineage) within swIAV H1 gene classification [12,13] (Table 1 and Figure S1).

### 2.2. Experimental Design

Specific pathogen-free (SPF) pigs were obtained from the biosecurity level 3 and air-filtrated pig herd of the French Agency for Food, Environmental, and Occupational Health and Safety (ANSES, Ploufragan, France). The experiment was performed in the ANSES facilities which have an agreement for animal experimentation, delivered by the *Direction Départementale de la Protection des Populations des Côtes d’Armor* (ANSES registration number C-22-745-1). The animal experiment protocol was approved by the French National Committee for Ethics in Animal Experimentation ANSES/ENVA/UPEC and authorized by the French Ministry for Research (approval No. 12/12/17-8).

Thirty-four week-old piglets were randomly allocated into six groups (Table 2). At five and eight weeks of age, three groups were vaccinated with a 2 mL intramuscular injection of vaccine Respiporc Flu^®^3. At nine weeks of age (day 0 (D0)), one unvaccinated group and one vaccinated group were inoculated intra-tracheally with 10^6^ TCID_50_ (50% tissue culture infectious dose) in a volume of 5 mL of the 415/11 strain (H1N2 and V+H1N2 groups, respectively). Two other unvaccinated and vaccinated groups were similarly inoculated with the variant 212/13 strain (H1N2var and V+H1N2var groups, respectively). The two last groups received 5 mL of Eagle’s Minimum Essential Medium (EMEM, Thermo Fisher Scientific, Waltham, MA, USA) (Control and V+Control groups).

### 2.3. Clinical Monitoring, Sampling and Necropsy

Rectal temperatures were recorded daily and the animals were weighed weekly. For each pig, other clinical signs were scored daily as follows (maximum score of 9): liveliness (normal = 0, reduced = 1, does not stand up = 2), appearance (normal = 0, emaciated = 1), respiration (normal = 0, increased frequency = 1, flank cut = 2), eye (normal = 0, red with clear secretions = 1, inflamed with turbid secretions = 2), and nasal discharge (absence = 0, clear discharge = 1, purulent flow = 2). In each room, coughs were counted for 15 min. every day.

Before vaccine injections at D-28 and D-7, and then once a week throughout the experiment, blood samples were collected, without additives or with heparin, to collect serum or peripheral blood mononuclear cells (PBMC), respectively, in order to monitor humoral and cellular immune responses. PBMC were isolated by Ficoll density gradient centrifugation while using LeucoSep tubes (Greiner Bio One, Les Ulis, France). Additional blood samples were collected at D1 and D2 in order to measure haptoglobin and cytokines in serum. Broncho-alveolar lavage fluids (BALF) were collected at D-5, D1, and D7 in order to evaluate the immune responses at the virus multiplication site. BALF were obtained by flushing the lungs with 2 × 20 mL of sterile phosphate buffered saline (PBS) while using a tracheal suction probe (Vygon, Ecouen, France). BALF collections were performed under general anesthesia obtained by intramuscular injection of 10 mg/kg Zoletil 100 (Virbac, Carros, France). The recovered BALF was then centrifuged to separate BALF-cells for flow cytometric analyses and the cell-free supernatant for antibody and cytokine quantification. Nasal swabs were taken daily for one week following the swIAV infection, and then every two days the following week, for virus excretion measurements. All of the samples were frozen at −20 °C, −70 °C or in liquid nitrogen until use with the exception of PBMC, which were analyzed extemporaneously.

All of the pigs were euthanized (anaesthesia with Zoletil 100 followed by bleeding) at D21 and post-mortem examinations were carried out. Pneumonia lesions were scored as previously described [14].

### 2.4. Viral Genome Quantification and Virus Isolation

The SwIAV M gene was detected and quantified in nasal swab supernatants by duplex M/β-actin RT-qPCR, as previously described [15]. Viral RNA amounts were expressed as the M gene copy number per 10^6^ copies of β-actin gene. As the presence of antibodies could decrease the infectiveness of virus particles; for vaccinated animals, virus propagation was attempted in MDCK cells from samples in which the amount of detected viral genome was quantifiable, according to standard protocol [11].

### 2.5. Haptoglobin and Cytokine Measurements

Haptoglobin was measured in serum while using a Phase Range Haptoglobin kit (Tridelta, Maynooth, Ireland). Porcine IL-6 and TNF-α were measured in serum and cell-free BALF, respectively, using ELISA commercial kits (Bio-techne, Minneapolis, MN, USA) and porcine IFN-α was quantified in cell-free BALF by an in-house ELISA [16].

### 2.6. Flow Cytometry Cellular Phenotype

The frozen BALF-cell samples were rapidly thawed at 37 °C and immediately washed in PBS. Subsequently, 5 × 10^5^ cells were transferred to 96-well plates and then double-stained with the following primary mouse monoclonal antibodies: RPE-coupled anti-swine CD172a, also identified as SWC3a (clone 74-22-15), FITC-coupled anti-swine CD203a, also identified as SWC9 (clone PM18-7) and unlabeled anti-swine SWC8 (clone MIL3) (all from BioRad, Hercules, CA, USA), or stained with the appropriate mouse isotype control (Life Technologies, Carlsbad, CA, USA or BioRad, Hercules, CA, USA). The unlabeled primary antibody was detected by a goat polyclonal secondary antibody FITC-conjugated anti-mouse IgM (BioRad, Hercules, CA, USA). Antibodies were used at the concentrations that were recommended by the manufacturers. The dead cells were excluded by a cell viability solution (BD Biosciences, San Jose, CA, USA), according to the manufacturer’s instructions. For each immunostaining, data from 30,000 events were acquired on a FC500 cytometer and analyzed with Kaluza 1.2 software (both Beckman Coulter, Fullerton, CA, USA).

### 2.7. Quantification of IFN-γ Secreting Cells

SwIAV specific IFN-γ secreting cells (IFNγ-SC) were quantified by enzyme-linked immunospot (ELISPOT) in PBMC. ELISPOT was performed in triplicate, as previously described [17]. Briefly, MultiScreen 96-well plates (Millipore, Burlington, MA, USA) were coated with 0.5 µg/well of purified mouse anti-swine IFN-γ antibody (clone P2G10, BD Biosciences, San Jose, CA, USA) overnight at 4 °C. For stimulation, 4 × 10^5^ PBMC were added to each well and incubated for 18 h at 37 °C, at a multiplicity of infection of 0.5, with either the virus strain inoculated to the sampled pigs, or with the other studied strain for evaluation of potential cross-reactivity. These stimulations were called here “homologous stimulation” and “heterologous stimulation”, respectively. After washing with PBS, IFN-γ was detected by the addition of 50 µL/well of biotinylated mouse anti-swine IFN-γ antibody (clone P2C11, BD Biosciences, San Jose, CA, USA) at 0.5 µg/mL for 2 h and then streptavidin-alkaline phosphatase (Thermo Fisher Scientific, Waltham, MA, USA) for 1 h at room temperature. Subsequently, spots that represent single IFNγ-SC developed after the addition of the substrate (BioRad, Hercules, CA, USA). The number of spots per well was counted using an ImmunoSpot S5 UV Analyzer (CTL, Shaker Heights, OH, USA). The number of IFNγ-SC was calculated by subtracting the number of potential non-specific spots that were obtained for the negative stimulation (cell culture medium) from the number of spots obtained for the viral stimulation, then expressed per 10^6^ PBMC. A positive control was performed by stimulating PBMC with phytohemagglutinin (Eurobio, Courtaboeuf, France).

### 2.8. Virus Neutralization Assays

Neutralizing antibodies (NA) targeting the H1_hu_N2 strain or the H1_hu_N2_Δ14–147_ strain were quantified in serum by a virus neutralization test, as previously described [18]. Briefly, sera were treated by receptor destroying enzyme (RDE) and adsorbed onto chicken erythrocytes in order to reduce non-specific reaction. Subsequently, they were two-fold serially diluted from 1/2 to 1/2048 and 50 μL of each dilution were incubated in duplicate in 96-well microtiter plates with 10^2^ TCID50/50μL of virus strain (415/11 or 212/13) for 1 h at 37 °C with rocking. Subsequently, the serum/virus mixture was inoculated into MDCK cells that were seeded the day before at 3 × 10^4^ cells per well, for 1.5 h at 37 °C with rocking. After two washings, the plates were incubated with 100 μL of an incubation buffer (EMEM, supplemented with penicillin, streptomycin and TPCK-treated trypsin (Worthington Biochemical Corporation, Lakewood, NJ, USA) at a final concentration of 2 μg/mL) for 72 h at 37 °C. The neutralizing antibody titer was determined as the reciprocal of the highest dilution of serum that prevents virus infection of the cell monolayer, as determined by the absence of cytopathic effect in half of the duplicate wells. The titers were log_2_ transformed in order to calculate the mean neutralizing titer of each group.

### 2.9. Haemagglutination Inhibition Assays

The haemagglutination inhibition (HI) test was performed to titrate antibodies directed against the HA protein in sera that were collected at D21, according to standard procedures [11]. Briefly, RDE-treated and erythrocyte-adsorbed sera were two-fold serially diluted from 1/10 to 1/5120 in 96-well plates and four haemagglutinating units (HAU) of virus were added to each well. The two challenge viruses and the three H1_hu_N2 reference strains were used as virus antigens. A 0.5% chicken red blood cells suspension was added and HI titers were read after 30 to 45 min. at room temperature. The titers were expressed as the reciprocal of the highest dilution inhibiting 4 HAU. HI titers equal to, or greater than, 10 were considered to be positive. For comparison, hyperimmune swine sera against the variant strain and the three reference antigens, produced in SPF pigs, as previously described [19], were included into the analysis as well as a serum obtained from a vaccinated sow [15]. This sow received five injections of Respiporc Flu^®^3 vaccine and the serum was collected four weeks after the last boost.

### 2.10. Statistical Analyses

For all data, non-parametric Kruskal–Wallis tests with Holm’s correction for pairwise comparisons were used in order to assess differences between groups. A Fisher test was also done to compare the number of RT-qPCR positive samples over the period D1 to D7 in both vaccinated/challenged groups. Correlation analyses between the clinical score at D1, duration of excretion, concentrations of haptoglobin at D2 and cytokines at D1, percentage of granulocytes (CD172a+SWC8+) and macrophages (CD172a+CD203a+) at D1 and D7, post-vaccinal responses (IFNγ-SC and NA) at D0, and number of IFNγ-SC and NA at D21 were performed using the Spearman rank correlation test. All of the statistical analyses were performed using R software (version 3.1.3). Differences were considered to be significant when *p*-values ≤ 0.05.

## 3. Results

### 3.1. Clinical Signs

At D1, animals from the H1N2var group obtained a significantly higher clinical score than those from the H1N2 group (mean scores of 1.6 ± 1.5 and 0.4 ± 0.9, respectively, *p* = 0.05). Four piglets out of the five in the H1N2var group showed clinical signs, such as increased respiratory rate, reduced liveliness, red eyes, or clear nasal discharge. In comparison, only 1/5 pig in the H1N2 group had an increased respiratory rate and reduced liveliness. In these two groups, all of the animals showed hyperthermia (rectal temperature > 40 °C) at D1 (41.1 ± 0.5 °C and 40.5 ± 0.5 °C on average for H1N2var and H1N2 groups, respectively) and a decrease in food consumption which resulted in reduced rate of weight gain during the post-inoculation week as compared to control animals (*p =* 0.01). Coughing was reported on D2, D5, and D6 in the H1N2var group, but not in the H1N2 group. At necropsy (D21), 5/5 animals from H1N2var group had lung lesions against only 1/5 animal in the H1N2 group. The mean macroscopic lung scores were of 1.8 ± 0.4/28 and 0.2 ± 0.4/28 for the H1N2var and H1N2 groups, respectively (*p* < 0.01).

By contrast, the vaccinated piglets were clinically protected after inoculation of the parental H1_hu_N2 virus, as no hyperthermia or other clinical signs were recorded in the V+H1N2 group. Vaccination also prevented the animals that were inoculated with the variant strain (V+H1N2var group) from developing clinical signs, except one of them who exhibited hyperthermia (40.2 °C) at D1. Both of the vaccinated-challenged groups showed similar weight gain to that of the control animals. No lung injury was observed in vaccinated animals at necropsy.

### 3.2. Virus Shedding

**Figure 1:**
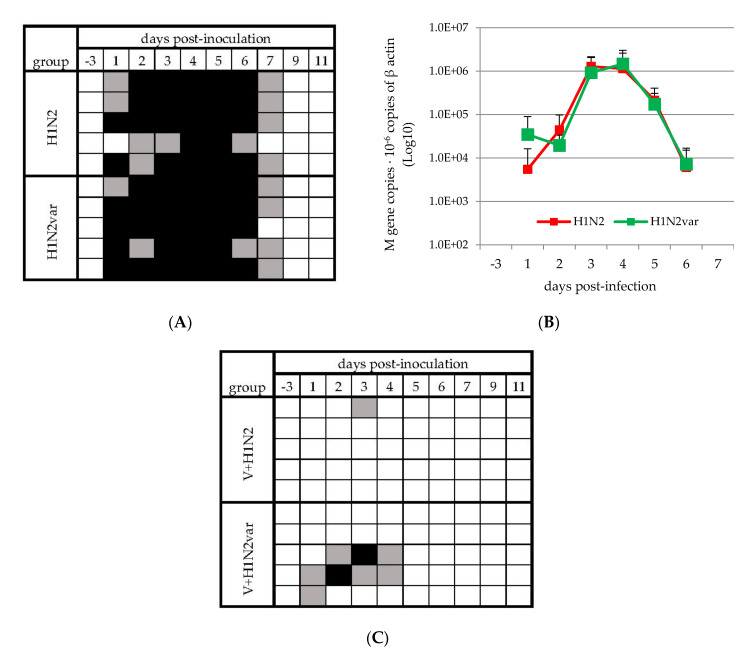
Virus excretion in nasal secretions. Individual results of M-gene RT-qPCR on nasal swab supernatants taken on unvaccinated (**A**) and vaccinated (**C**) pigs. Black and grey squares indicate the detection of quantifiable and not quantifiable (under the quantification limit threshold of 2 × 10^2^ copies of M gene) swIAV genome, respectively. White squares indicate that the virus genome was not detected. (**B**) Average of viral RNA amounts obtained in unvaccinated groups, including quantifiable RNA, non-quantifiable RNA, and negative samples (the value 0 has been assigned in the latter two cases).

All unvaccinated but inoculated animals shed virus between D1 and D7, regardless of the inoculated strain (Figure 1A). The highest genomic loads were measured at D3–D4, with similar amounts in both the H1N2 and H1N2var groups (Figure 1B).

In the vaccinated V+H1N2 group, virus shedding was strongly reduced, almost totally, as compared to the unvaccinated H1N2 group, as the virus genome was only transiently and slightly detected in one animal at D3 with a M-gene amount that was below the quantification limit threshold of the RT-qPCR (<2 × 10^2^ copies of M gene) (Figure 1C).

By contrast, vaccination was less effective in preventing virus shedding in the V+H1N2var group, since the virus genome was detected in 3/5 animals between D1 and D4 (Figure 1C). Thus, the number of samples in which the virus genome was detected was significantly higher in this group than in the V+H1N2 group (*p* = 0.03). Moreover, the amount of virus genome that was detected in two of these positive samples from V+H1N2var group was high enough to be quantified (4.23 × 10^4^ copies at D2 and 1.42 × 10^4^ copies at D3, respectively) and infectious viral particles were re-isolated by propagation in cell culture from these samples.

### 3.3. Quantification of Haptoglobin and Cytokines in Sera or BALF

**Figure 2:**
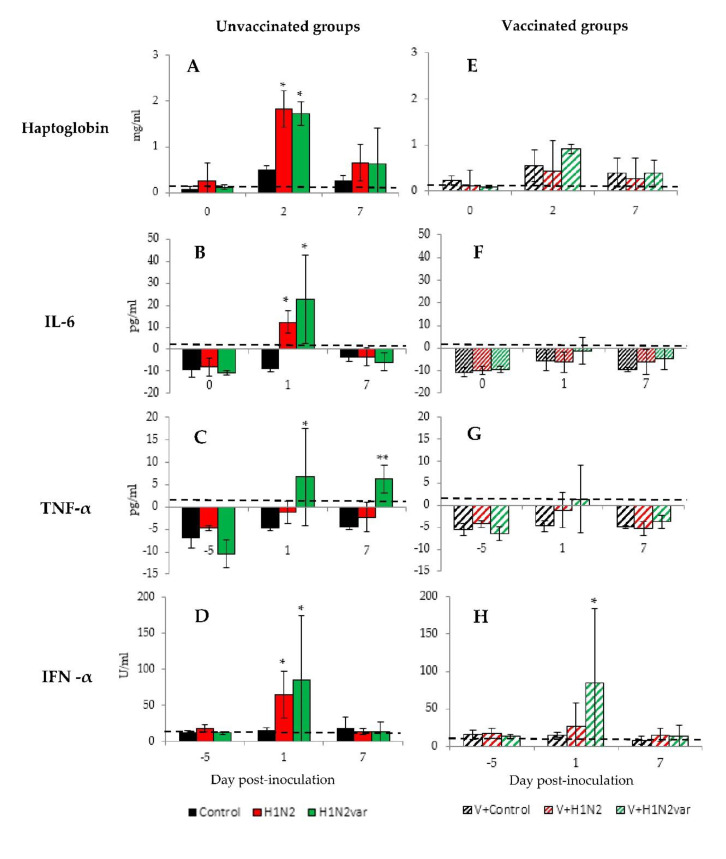
Concentrations of haptoglobin, pro-inflammatory cytokines and interferon-α in serum and broncho-alveolar lavage fluids (BALF) following swine influenza A viruses (swIAV) infections. Mean ± standard deviation of concentrations of haptoglobin (**A**,**E**) and IL-6 (**B**,**F**) in sera, and TNF-α (**C**,**G**) and IFN-α (**D**,**H**) in cell-free BALF for unvaccinated groups (full bar) or vaccinated groups (hatched bar), inoculated with the 415/11 strain (red), the 212/13 strain (green) or with culture medium (black). The limits of detection for these methods were 0.05 mg/mL for haptoglobin, 2.03 pg/mL for IL-6, 3.7 pg/mL for TNF-α and 11.5 U/mL for IFN-α and are represented by the hatched line. * indicates that the group is significantly different (*p ≤* 0.05) from its Control group. ** indicates that the group is significantly different (*p ≤* 0.05) from its Control group and from the group inoculated with the other virus strain.

Regardless the swIAV strain that they were inoculated with, all unvaccinated animals showed an increase in concentrations of haptoglobin and pro-inflammatory cytokine IL-6 in serum after the challenge (Figure 2A,B), revealing inflammatory conditions in both unvaccinated groups. However, despite large individual variations, TNF-α was also significantly detected at D1 and D7 in BALF of piglets from H1N2var group, but not the H1N2 group (Figure 2C). Regarding the anti-viral response, average concentrations of IFN-α in BALF increased significantly at D1, similarly in both H1N2 and H1N2var groups (*p* ≤ 0.05) (Figure 2D).

By contrast, in V+H1N2 animals, the concentrations in haptoglobin and pro-inflammatory cytokines remained close to the basal levels measured in Control pigs (Figure 2E–G) and IFN-α was not detected (Figure 2H), which indicated that these piglets did not develop any inflammatory or antiviral responses following infection. In the V+H1N2var group, the mean concentrations in haptoglobin, IL-6, and TNF-α have not been changed as compared to the Control group either, but individual responses to the infection appeared to be heterogeneous, as shown by the large standard deviations. At the individual level, one animal exhibited significantly higher concentrations in IL-6 and TNF-α in BALF at D1 as compared to the Control animals. In this V+H1N2var group, a significant increase in IFN-α amount in BALF was detected at D1 (*p =* 0.05).

### 3.4. Phenotype of Myeloid Cells Collected in Broncho-Alveolar Fluids

**Figure 3:**
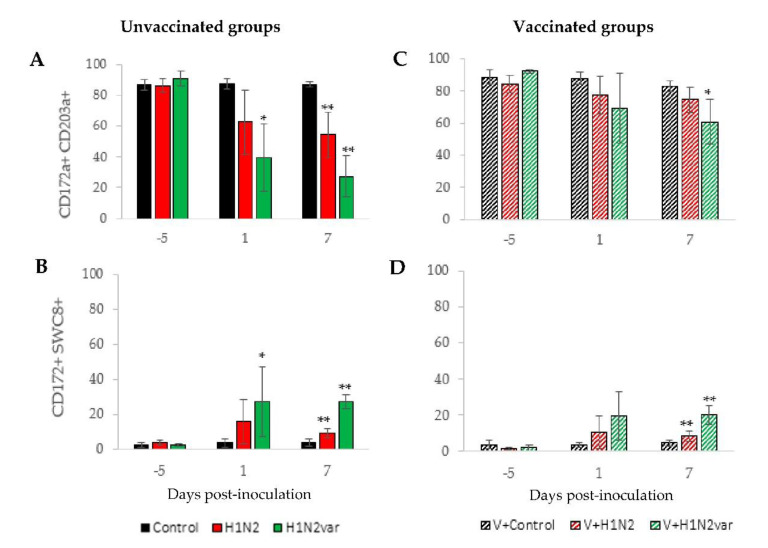
Phenotypic analysis of cells from BALF. Proportion of CD172a+, CD203a+ (**A**,**C**), and CD172a+SWC8+ (**B**,**D**) cells in BALF collected at D-5, D1, and D7 in unvaccinated animals (full bar) or vaccinated animals (hatched bar) and inoculated with the 415/11 strain (red), the 212/13 strain (green) or culture medium (black). Results are expressed as percentage (±standard deviation) of cells expressing the molecule among the total population of viable CD172a+ myeloid cells in BALF. * indicates that the group is significantly different (*p ≤* 0.05) from its Control group. ** indicates that group is significantly different (*p ≤* 0.05) from its Control group and from the group inoculated with the other virus strain.

Before virus inoculations, myeloid cells (CD172a+) that were collected in BALF were mainly pulmonary macrophages (CD172a+CD203a+ cells), which counted for 84 to 92% of this fraction, depending on the group (Figure 3A,C). Granulocytes (CD172a+SWC8+ cells) counted for 2 to 4% only (Figure 3B,D).

After virus inoculations in unvaccinated groups, a decrease in the proportion of macrophages and an increase in the percentage of granulocytes were observed in both H1N2 and H1N2var groups (Figure 3A,B). However, these modifications occurred faster in the H1N2var group, since only the H1N2var group was significantly different from the Control group at D1. Moreover, the afflux of granulocytes in lungs was more pronounced in the case of infection with the variant, as revealed by relative proportions that were measured at D7. At that time, macrophages and granulocytes represented 27.3% (±13.3%) and 27% (±3.9%) of myeloid cells in the H1N2var group, respectively, whereas 54.5% (±14.8%) and 9.1% (±2.8%) in the H1N2 group, respectively.

In vaccinated groups, both of the swIAV infections also induced an afflux of granulocytes concurrently to a decrease in macrophage’s proportion in BALF-cell fractions at D7, but to a lesser extent than in unvaccinated groups (Figure 3B,D). Thus, macrophages and granulocytes represented 74.4% (±7.5%) and 8.5% (±3%) of myeloid cells, respectively, in the V+H1N2 group, and 60.5% (±14.1%) and 20% (±5.2%), respectively, in the V+H1N2var group.

### 3.5. Evaluation of the Cellular Adaptive Immune Response in Peripheral Blood Mononuclear Cells

**Figure 4:**
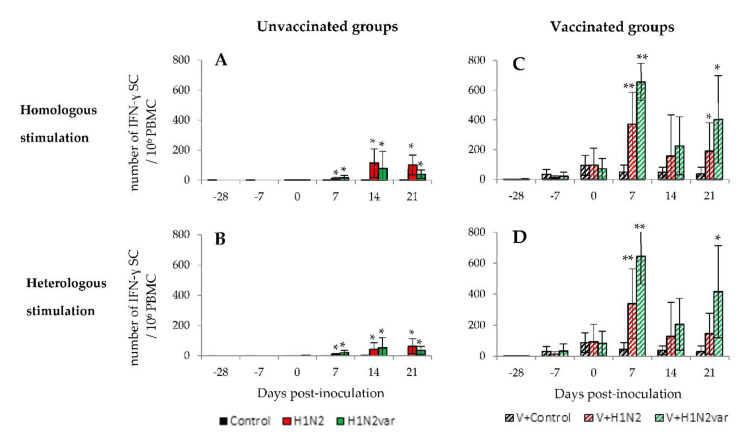
Evolution of cellular adaptive immune responses over time**.** Means (±standard deviation) of counts of IFN-γ secreting cells (IFNγ-SC) in unvaccinated (**A**,**B**) and vaccinated (**C**,**D**) animals obtained after a homologous (**A**,**C**) or heterologous (**B**,**D**) stimulation of peripheral blood mononuclear cells (PBMC) collected at D-28, D-7, D0, D7, D14, and D21. For the Control group, results obtained after a stimulation with the 415/11 and the 212/13 strains were averaged and represented in A and B. For the V+Control group, the results obtained after a stimulation with the 415/11 are presented in C and those obtained after a stimulation with the 212/13 strain are presented in D. * indicates that the group is significantly different (*p ≤* 0.05) from its control group. ** indicates that the group is significantly different (*p ≤* 0.05) from its control group and from the group inoculated with the other virus.

In unvaccinated animals, no cellular response was detected in the Control group throughout the study (only 0 to 3 IFNγ-SC/10^6^ PBMC for each pig), either after stimulation with the 415/11 strain or with the 212/13 strain. Therefore, for this group, data that were obtained with both stimulations were averaged and represented in Figure 4A,B. SwIAV-specific IFNɣ-SC were detected in both H1N2 and H1N2var groups at D7 in response to an homologous stimulation with the virus that was inoculated to the pigs (*p* < 0.01) and their numbers reached a plateau at D14 (Figure 4A). No significant differences were observed between these two groups (*p* > 0.05). The same results were obtained after a heterologous stimulation of PBMC, which indicated a cross-reactivity of IFNγ-SC, i.e., these cells were capable of being stimulated by either of the two studied viruses (Figure 4B).

In vaccinated animals, a cell-mediated immune response was evidenced following the vaccination booster, as shown by the numbers of IFNγ-SC that were measured at D0 in the V+Control group after stimulation with the 415/11 strain (Figure 4C). These post-vaccine IFNɣ-SC were similarly activated by the variant 212/13 strain (Figure 4D). After virus inoculations, the IFN-ɣ responses increased in both groups after a homologous stimulation, although more strongly in the V+H1N2var group than in the V+H1N2 group at D7 (*p* = 0.02), before decreasing in both groups at D14 and then increasing slightly at D21 (Figure 4C). The same variations in numbers of IFNɣ-SC were measured after PBMC stimulation with the other virus than the one that was inoculated to the tested animals, except for the V+H1N2 group, for which no slight increase was observed at D21 (Figure 4D).

### 3.6. Evaluation of the Neutralizing Immune Response in Sera

**Figure 5:**
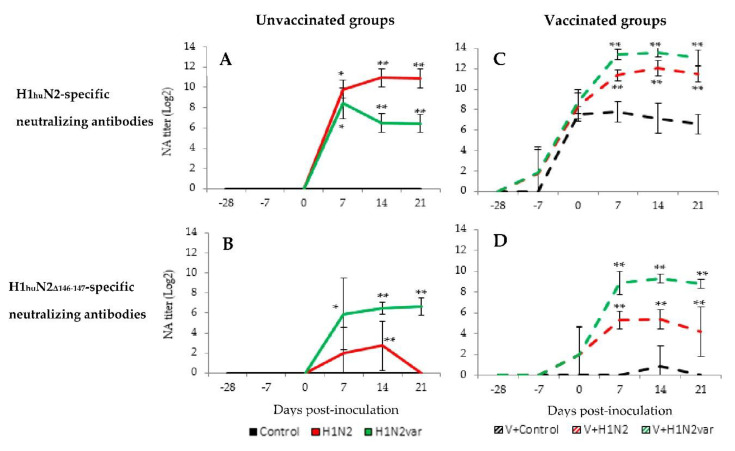
Evolution of humoral adaptive immune responses over time. Quantification of neutralizing antibodies (NA) in sera by neutralization test with the 415/11 strain (**A**,**C**) or with the 212/13 strain (**B**,**D**). Presented results are means ± standard deviations of log_2_ transformed neutralizing titers for unvaccinated (solid lines) and vaccinated (dotted lines) groups inoculated with the 415/11 strain (red), the 212/13 strain (green) or culture medium (black). * indicates that the group is significantly different (*p ≤* 0.05) from its Control group. ** indicates that the group is significantly different (*p ≤* 0.05) from its Control group and from the group inoculated with the other virus.

In unvaccinated animals, no swIAV-specific neutralizing antibodies were detected in the Control group throughout the study (Figure 5A,B). H1_hu_N2-specific neutralizing antibodies were similarly detected in H1N2 and H1N2var groups as early as D7, but at D14 and D21, the quantity of neutralizing antibodies was higher in the H1N2 group than in the H1N2var group (Figure 5A). Indeed, the mean neutralizing titers reached 10.9 log_2_ at D14 and D21 in the H1N2 group against 6.5 and 6.4 log_2_ at D14 and D21, respectively, in the H1N2var group. Regarding the H1_hu_N2_Δ14–147_-specific neutralizing antibodies, it appeared that very few post-infectious antibodies in the H1N2 group were able to neutralize the variant 212/13 strain, since the difference from the Control group was observed only at D14 (Figure 5B). On the contrary, in the H1N2var group, the mean titer of H1_hu_N2_Δ14–147_-specific neutralizing antibodies increased and reached a plateau at D14 and D21, with mean titers of 6.5 and 6.6 log_2_, respectively, which was significantly different from the H1N2 group.

In all of the vaccinated animals, post-vaccine antibodies present in sera collected at D0 before inoculations showed their ability to neutralize the parental 415/11 virus, which is genetically close to the vaccine antigen, since the mean titer obtained by all vaccinated animals was 8.2 log_2_ (Figure 5C). On the other hand, post-vaccine antibodies appeared to be less able to neutralize the variant 212/13 virus, as the mean neutralizing antibody titers that were obtained by all vaccinated animals was only 1.3 log_2_ at D0 (Figure 5D). Inoculations of vaccinated animals induced a new production of neutralizing antibodies against 415/11 and 212/13 strains_,_ since the mean titers of both V+H1N2 and V+H1N2var groups were significantly different from those that were measured in the V+Control group at D7, D14, and D21 (Figure 5C,D). The humoral response boost appeared to be greater in the V+H1N2var group than in V+H1N2 group (*p* < 0.01 at D7, D14, and D21).

### 3.7. Evaluation of Cross-Reactive Anti-H1_hu_ Antibodies in Sera at D21

Table 3 presents the results of haemagglutination inhibition assays. In the H1N2 group, the mean HI titer of sera taken at D21 reached 422.2 (320–640) when tested with the challenge 415/11 strain, whereas it was only 20.0 (10–40) with the 212/13 antigen. These results were concordant with those that were obtained with the H1_hu_N2 reference antigens, which reacted better with the recent H1_hu_N2 antigens—214/06, 113/06, and 415/11—than with the variant 212/13 strain. On the contrary, in the H1N2var group, the mean HI titer only reached 40.0 (20–80) with the 415/11 antigen, but 211.1 (160–320) with the challenge variant strain, which is congruent with the results obtained with the reference serum 212/13. All of the sera taken from H1N2 and H1N2var groups at D21, as reference sera 214/06, 113/06, and 212/13, showed a low reaction with the common ancestral Scotland/94 virus strain.

The sera from the V+Control group did not exhibit any HI titer when tested with the Scotland/94 antigen, but positive HI titers up to 40 were measured when tested with the three more recent H1_hu_N2 strains, including the 415/11. By contrast, they were found to be negative when tested with the variant strain. The reference serum from the vaccinated sow confirmed stronger reactions with the more recent H1_hu_N2 antigens than with the ancestral Scotland/94 strain or the variant antigen. In the V+H1N2 and V+H1N2var groups, inoculation with 415/11 or 212/13, respectively, boosted HI responses initiated following vaccination, since HI titers significantly higher than in the V+Control group were obtained, whatever the tested antigens. This boost was greater (with at least a threefold difference) in V+H1N2var group than in V+H1N2 group. No difference was observed between the H1N2 and V+H1N2 groups or between the H1N2var and V+H1N2var groups, with the 415/11 or 212/13 antigen, respectively, but a stronger reaction was observed in vaccinated animals than in unvaccinated animals with the virus that was not inoculated into the sampled pigs.

### 3.8. Correlation Analyses

Correlation analysis performed with data from unvaccinated animals indicated that the H1N2 virus shedding was positively correlated with serum concentrations in haptoglobin, IL-6, TNF-α, and IFN-α, as well as with the percentage of granulocytes in lungs at D7 and amounts of IFNɣ-SC and H1_hu_N2-specific neutralizing antibodies in blood (Table 4). By contrast, it was negatively correlated with the percentage of macrophages in lungs at D7. All of these parameters were similarly correlated in the case of infection with the 212/13 virus (Table 4). However, after infection with the variant, additional positive correlations were found. Indeed, the clinical score of animals was related to the virus excretion, the levels of TNF-α and IFN-α at D1, the percentages of granulocytes at D1 and D7, as well as the adaptive immune response implemented at D21 (Table 5). The virus excretion was also correlated to the amount of granulocytes at D1 and the variant-specific neutralizing antibody titer at D21. Both clinical score and excretion were negatively correlated with the percentages of macrophages at D1.

Correlation analysis that was performed with data from vaccinated animals infected with H1_hu_N2 indicated that the amount of post-vaccination antibodies were negatively correlated with the viral shedding and the inflammatory (haptoglobin, IL-6) and antiviral (IFN-α) responses (Table 6). On the other hand, a positive correlation was observed between the humoral response that was induced by vaccination at D0 and the proportion of macrophages among myeloid cells at D7 and also with the level of the H1_hu_N2_Δ14–147_-specific neutralizing antibodies at D21. However, no relationship was observed between the post-vaccine antibody levels and the proportions of granulocytes and macrophages at D1 or the cellular response and specific-H1_hu_N2 neutralizing antibody levels at D21. In the case of vaccinated animals that were infected with the 212/13 strain, a negative correlation was observed between the immune post-vaccine responses (cellular and humoral) at D0 and the clinical score, the virus shedding, the IL-6 concentration, and the percentage of granulocytes at D7 (Table 7). Post-vaccine responses were positively correlated with the proportion of macrophages at D7 and the adaptive immune responses at D21. There was no association between post-vaccine responses at D0 and haptoglobin, TNF-α, IFN-α levels, and percentages of granulocytes and macrophages at D1.

## 4. Discussion

The first objective of this work was to study the clinical, virological, and immunological responses of pigs infected with the variant 212/13 virus strain (H1N2var group), as compared to those that were observed in pigs infected with the parental 415/11 virus strain (H1N2 group). The variant appeared to be more pathogenic than the parental virus, since the animals in the H1N2var group showed overall more clinical signs than those in the H1N2 group. The severity of clinical signs was correlated with the level of the pro-inflammatory response, notably the production of TNF-α and a huge afflux of granulocytes into the lung. The TNF-α, which is primarily produced by macrophages and monocytes, is an important cytokine playing multiple roles in damage and inflammation of the lungs [20]. Among its numerous actions, it can be cited that TNF-α is a neutrophil and eosinophil chemoattractant; it stimulates the leukocyte accumulation, proliferation, and differentiation at the site of infection and contributes to the induction of oxidative stress [20]. This could explain why all animals in the H1N2var group still presented with lung lesions three weeks after inoculation, but not the animals in the H1N2 group. The link between the production of TNF-α and the infection severity we observed here is consistent with our previous work studying co-infection with *Mycoplasma hyopneumoniae* and a swIAV of the H1_av_N1 subtype, in which we showed that severe lung lesions were associated with a high production of TNF-α [21]. Although no difference was observed between both H1N2 and H1N2var inoculated groups regarding the levels of nasal virus shedding, it cannot be excluded that greater respiratory disorders in H1N2var animals may facilitate variant virus spreading within a group. Whereas, coughing and sneezing are not a prerequisite for generating fine aerosol droplets, which can be produced by breathing alone, they could nevertheless contribute to expanded surface contamination and the airborne transmission of influenza A virus [22,23].

Regarding adaptive responses, antibodies that were produced following 415/11 infection were not able to neutralize the variant 212/13 virus, which confirmed an antigenic distance between both viruses, probably in line with mutations in H1 antigenic sites (Figure S1), as immunodominant epitopes that are targeted by the humoral adaptive immune response are present on HA surface protein [24]. On the contrary, the same quantities of IFN-ɣ secretory cells were obtained, regardless of the strain used to stimulate PBMC from infected animals, indicating that T cells recognized epitopes common to both viruses. Such immunodominant epitopes that are linked to cellular responses are typically found in internal viral proteins—matrix 1 (M1), nucleoprotein (NP), polymerase acidic subunit (PA), and polymerase basic subunit 1 (PB1)—which are quite conserved between swine influenza A viruses of same viral origin, allowing for T cells to cross-react with antigenic variants within the same swIAV subtype and, to a lesser extent, with other swIAV subtypes [10,24,25,26]. In silico comparison of whole genome sequences of 415/11 and 212/13 strains showed mutations on NP (D112E, I232T, E292G), PB1 (V591I), M2 (T11I, R18K, D21G, I28T et I39L), and PB2 (T559N) in the variant strain, at sites that are described as epitopes for T cells (www.iedb.org), but these mutations did not seem to be sufficient to alter cellular responses that are common to the parental strain.

Further studies would be necessary to investigate the mechanisms that underlie the marked inflammation induced by the variant and determine which genetic modifications were responsible for that. Amino acid deletions in the RBS may have an impact on the receptor specificity, which, consequently, could modify the cellular tropism in the respiratory tract and contribute to pathogenicity [27]. Additional mutations that were observed in NA may have been introduced in response to the modifications in HA to re-establish the HA/NA functional balance, leading to a modification of virulence. Intriguingly, deletions 146 and/or 147 have been identified in H1 of many swIAV virus strains worldwide [3,5,6,7,8,9], whereas these viruses were of different viral origins and they belonged to different lineages. This genetic evolution would certainly tend to bring an advantage for the virus; in vitro analyses using recombinant viruses produced by reverse genetics would allow for a better understanding of this phenomenon.

The second objective of this study was to know whether the vaccine that is currently available in Europe against H1_hu_N2 viruses is able to protect the animals from an infection with the variant H1_hu_N2_Δ14–147_, as compared to the parental virus. In our experimental conditions, vaccinated piglets were clinically protected against both the parental and the variant viruses. Moreover, vaccination reduced the viral shedding in both groups. However, it can be noted that, even if the viral shedding was reduced in the V+H1N2var group, it was not completely inhibited, as observed in the V+H1N2 group. In addition, some V+H1N2var animals remained potentially infectious, since live virus was isolated from their nasal secretions. One of them also presented signs of inflammation, i.e., peaks of pro-inflammatory cytokines and a high influx of granulocytes, following 212/13 inoculation. 

Vaccination established a humoral response at D0. However, post-vaccine antibodies were able to neutralize the 415/11 virus, but very little the 212/13 virus. This lower effectiveness of the vaccine response towards the variant virus was confirmed by the lack of detection of anti-HA antibodies recognizing the 212/13 antigen in the V+Control group four weeks after vaccination. Thus, at the time of the challenge, the humoral response induced by the vaccination would have failed in protecting the V+H1N2var animals, since anti-HA antibodies constitute the major neutralizing response against IAV infection [28]. However, post-vaccine antibodies might have been produced against the N2 protein of the vaccine antigen. Anti-NA antibodies are not considered to be neutralizing, but they may interfere with the virus release from the cell surface or participate in antibody dependent cell-mediated cytotoxicity. Thus, such anti-N2 antibodies could have potentially played a role in some cross-protective reaction.

Contrary to the humoral response, the cell-mediated response was detected at D0 in all vaccinated groups after stimulation with both 415/11 and 212/13 strains. Although a study demonstrated that an inactivated swIAV vaccine stimulated cellular immune responses, including the CD8+ T cell subset in pigs [29], this type of vaccine is better known to induce only a limited cellular immunity, because killed viruses do not enter the endogenous pathway of antigen presentation and, consequently, are unable to activate CD8+ T cells [10,28,30]. In the present study, it cannot be determined whether swIAV-specific IFN-γ secreting cells measured at D0 reflected the activity of CD4+ T helper cells or CD8+ cytotoxic T cells. Either way, it can be assumed that V+H1N2var animals were mainly protected against the variant virus through their cellular immunity and, potentially, by antibodies targeting proteins other than the HA. It can be hypothesized that these protective immune responses would not have been as rapid and effective as anti-HA antibodies in neutralizing the variant virus as soon as it enters the host, giving it time to multiply and be excreted by some vaccinated animals. 

Finally, it can be noted that the boost of the adaptive responses—IFN-γ secreting cells, neutralizing, and HI antibodies—was greater when the vaccinated animals were challenged with the variant virus than with the parental virus. The fact that consecutive infections or vaccinations with antigenically distinct viruses favor the antibody response to the virus strain encountered first, with sometimes an impairment of the antibody response to the second strain, is called “back-boosting” or “original antigenic sin” [31]. For example, it has been shown that inoculations of pigs with H1_av_N1 then with H1_hu_N2 were followed by an important rise in H1_av_N1-specific antibodies after H1_hu_N2 inoculation [32]. Similarly, a heterologous prime-boost vaccination of pigs with two antigenically distinct H3N2 enhanced antibody responses against both of the vaccine strains [33]. Otherwise, the immunization of pigs with an inactivated adjuvanted monovalent vaccine followed by a challenge with an antigenically divergent strain of the same subtype can have an adverse effect. Indeed, pigs can develop enhanced respiratory pathology due to a strong stimulation of non-neutralizing antibody response [28]. This phenomenon, which is called vaccine-associated enhanced respiratory disease (VAERD), was not observed in the present study with the trivalent vaccine.

The objective of swine influenza vaccination using inactivated antigens is to reduce clinical signs and viral lung load after infection, as compared to unvaccinated animals [34]. Thus, the data that were obtained in V+H1N2 and V+H1N2var groups were in accordance with the expected benefits of the vaccine in the field. However, the outcomes of infections with the variant and the parental viruses in vaccinated animals were substantially different, with a less-than-optimal immunological vaccine response against the variant and infectious particles of this virus excreted by vaccinated piglets. These results would need to be confirmed in vaccinated breeder herds, as they are of the most concerned with respect to swine influenza vaccination in field, but it can be assumed that the variant virus could more easily spread among vaccinated animals. A future experimental study involving naive animals that are placed in indirect contact with infected animals—vaccinated or not—would make it possible to verify this hypothesis and complete our knowledge of the dynamics and impact of this viral variant on pig farming. If confirmed, escaping vaccine immunity could give an advantage to the variant that could possibly become enzootic. In France, even though the proportion of H1_hu_N2_Δ14–147_ strains among the H1_hu_N2 viruses has decreased since 2015, they are still detected every year, which confirms the virus maintenance within the pig population [3]. Therefore, it is necessary to follow further genetic and antigenic evolutions of this variant that diverges from the vaccine antigen. Strains that are included in swine influenza vaccine are rarely updated [28], since antigenic drift of influenza A viruses has been considered to be much slower in swine than in human [35]. However, in recent years, the diversity of antigenic variant swIAV has been steadily increasing in pigs, because of antigenic shift or drift [36,37,38]. Thus, the question of updating the vaccine antigens and/or change the vaccine strategy (e.g., live attenuated influenza virus vaccine) to better adapt to the field situations can arise.

## Figures and Tables

**Table 1:** H1 genotypes of H1N2 virus strains used in the study.

Strain	GenBank Accession Number of the HA Gene ^1^	H1 Clade	Used as
A/Sw/Bakum/1832/2000 (Bakum/00)	GQ161104	1B.1.2.1	vaccine antigen
A/Sw/France/Ille et Vilaine-0415/2011 (415/11)	KR699790	1B.1.2.3	challenge strain (H1_hu_N2)
A/Sw/France/22-130212/2013 (212/13)	KJ128323	1B.1.2.3 Δ146–147	challenge strain (H1_hu_N2_Δ146–147_)
A/Sw/Scotland/410440/94 (Scotland/94)	AF085413	1B.1	reference strain for HI test
A/Sw/Cotes d’Armor/0214/06 (214/06)	AM777812	1B.1.1	reference strain for HI test
A/Sw/Cotes d’Armor/0113/06 (113/06)	AM503902	1B.1.2.3	reference strain for HI test

^1^ GenBank accession number of the other genes are available upon request.

**Table 2:** Experimental design.

Group ID	Vaccination with Respiporc Flu^®^3 at 5 and 8 Weeks of Age (D-28 and D-7, Respectively)	Virus Strain Intra-Tracheally Inoculated at 9 Weeks of Age (D0)
H1N2	no	415/11 ^1^
H1N2var	no	212/13 ^2^
Control	no	EMEM ^3^
V+H1N2	yes	415/11 ^1^
V+H1N2var	yes	212/13 ^2^
V+Control	yes	EMEM ^3^

^1^ 415/11 = A/Sw/France/Ille et Vilaine-0415/2011 (H1_hu_N2); ^2^ 212/13 = A/Sw/France/22-130212/2013 (H1_hu_N2_Δ146–147_); ^3^ EMEM = Eagle’s Minimum Essential Medium.

**Table 3:** Geometric mean of haemagglutination-inhibition (HI) titers of sera taken at D21 in each experimental group towards three reference H1_hu_N2 antigens and the challenged strains. Ranges of HI titers are reported between brackets. Hyperimmune sera produced in SPF pigs and a serum taken from a vaccinated sow were included in the assay as controls. A serum sample was positive towards a given antigen when HI titer ≥ 10. Different letters indicate a significant difference between groups for a given antigen (column analysis), or a significant difference between antigens for a given group (row analysis) with *p ≤* 0.05.

		H1N2 Virus Strains				
		Reference Antigens			Challenged Strains	
Serum		A/Sw/Scotland/410440/94 (H1_hu_N2—Clade 1.B.1)	A/Sw/Cotes d’Armor/0214/06 (H1_hu_N2—Clade 1B.1.1)	A/Sw/Cotes d’Armor/0113/06 (H1_hu_N2—Clade 1B.1.2.3)	A/Sw/France/Ille et Vilaine-0415/2011 (H1_hu_N2—Clade 1B.1.2.3)	A/Sw/France/22-130212/2013 (H1_hu_N2_Δ14–147—_Clade 1B.1.2.3 Δ14–147)
**Reference** **Sera**	A/Sw/Scotland/410440/94 (H1_hu_N2)	640	640	640	1280	20
	A/Sw/Cotes d’Armor/0214/06 (H1_hu_N2)	20	1280	320	640	80
	A/Sw/Cotes d’Armor/0113/06 (H1_hu_N2)	10	80	320	160	10
	A/Sw/France/22-130212/2013 (H1_hu_N2 _Δ14–147_)	10	160	80	160	640
	Serum from a vaccinated sow [15]	40	160	80	160	40
**H1N2 group**		6.3 ^a^ (<10–10)	80.0 ^b^ (40–160)	91.9 ^b^ (40–160)	422.2 ^c^ (320–640)	20.0 ^d^ (10–40)
**H1N2var group**		1.0 ^e^ (<10)	45.9 ^f^ (20–80)	13.2 ^g^ (10–20)	40.0 ^f^ (20–80)	211.1 ^h^ (160–320)
**V+Control group**		1.0 ^e,i^ (<10)	20.0 ^j^ (10–40)	9.6 ^g,j^ (<10–40)	26.4 ^f,j^ (20–40)	1.0 ^i^ (<10)
**V+H1N2 group**		26.4 ^k^ (10–40)	278.6 ^l^ (160–640)	242.5 ^l^ (80–320)	367.6 ^c,l^ (160–640)	45.9 ^k^ (20–80)
**V+H1N2var group**		91.9 ^m^ (80–160)	2228.6 ^n^ (1280-≥5120)	844.5 ^n,o^ (320–2560)	1280.0 ^n,o^ (640–2560)	422.2 ^h,m,o^ (160–1280)

Titers < 10 were assigned a value of 1 for calculation of geometric mean HI titer and HI titer ≥ 5120 was assigned a value of 5120. Grey = homologous reaction.

**Table 4:** Correlation analysis between parameters obtained in pigs infected with the 415/11 strain. Spearman’s correlation coefficients between clinical score at D1, duration of viral excretion, concentrations of haptoglobin in serum at D2 and cytokines in serum or BALF at D1, percentage of granulocytes (CD172a+SWC8+), and macrophages (CD172a+CD203a+) in BALF at D1 and D7, number of IFNγ-SC within PBMC and neutralizing antibody (NA) titers in serum at D21. Analysis performed with data that were obtained in unvaccinated H1N2 and Control groups.

	Excretion	Haptoglobin	IL-6	TNF-α	IFN-α	Granulocytes D1	Macrophages D1	Granulocytes D7	Macrophages D7	IFNγ-SC D21	H1_hu_N2-Specific NA D21	H1_hu_N2_Δ14–147_-Specific NA D21
clinical score	0.38	0.41	0.28	0.41	0.53	0.52	−0.41	0.52	−0.29	0.41	0.25	na
excretion		0.87	0.73	0.69	0.77	0.58	−0.40	0.73	−0.77	0.85	0.91	na
haptoglobin			0.83	0.55	0.54	0.64	−0.26	0.57	−0.81	0.79	0.71	na
IL-6				0.54	0.44	0.62	−0.47	0.68	−0.95	0.75	0.65	na
TNF-α					0.76	0.82	−0.81	0.69	−0.62	0.77	0.84	na
IFN-α						0.69	−0.69	0.92	−0.60	0.81	0.83	na
granulocytes D1							−0.79	0.71	−0.64	0.84	0.70	na
macrophages D1								−0.71	0.47	−0.71	−0.62	na
granulocytes D7									−0.77	0.82	0.79	na
macrophages D7										−0.80	−0.72	na
IFNγ-SC D21											0.87	na
H1_hu_N2-specific NA D21												na

na = not applicable. Grey cells indicate that the correlation is significant at *p* ≤ 0.05.

**Table 5:** Correlation analysis between parameters obtained in pigs infected with the 212/13 strain. Spearman’s correlation coefficients between clinical score at D1, duration of viral excretion, concentrations of haptoglobin at D2 and cytokines at D1, percentage of granulocytes (CD172a+SWC8+) and macrophages (CD172a+CD203+) at D1 and D7, number of IFNγ-SC, and neutralizing antibody (NA) at D21. Analysis performed with data that were obtained in unvaccinated H1N2var and Control groups. Grey cells indicate that the correlation is significant at *p* ≤ 0.05.

	Excretion	Haptoglobin	IL-6	TNF-α	IFN-α	Granulocytes D1	Macrophages D1	Granulocytes D7	Macrophages D7	IFNγ-SC D21	H1_hu_N2-Specific NA D21	H1_hu_N2_Δ146__-147_-Specific NA D21
clinical score	0.76	0.59	0.61	0.75	0.67	0.77	−0.83	0.83	−0.84	0.65	0.69	0.80
excretion		0.90	0.83	0.83	0.73	0.90	−0.87	0.87	−0.80	0.78	0.93	0.84
haptoglobin			0.74	0.83	0.61	0.90	−0.71	0.67	−0.68	0.72	0.79	0.79
IL-6				0.82	0.65	0.66	−0.65	0.64	−0.87	0.89	0.83	0.68
TNF-α					0.79	0.76	−0.77	0.61	−0.82	0.73	0.66	0.75
IFN-α						0.54	−0.60	0.69	−0.70	0.66	0.59	0.68
granulocytes D1							−0.88	0.81	−0.79	0.74	0.85	0.80
macrophages D1								−0.89	0.81	−0.74	−0.84	−0.84
granulocytes D7									−0.82	0.73	0.84	0.84
macrophages D7										−0.93	−0.82	−0.80
IFNγ-SC D21											0.85	0.79
H1_hu_N2-specific NA D21												0.76

Grey cells indicate that the correlation is significant at *p* ≤ 0.05.

**Table 6:** Correlation analysis between parameters obtained in vaccinated piglets infected with the parental 415/11 strain. Spearman’s correlation coefficients between clinical score at D1, duration of viral excretion, concentrations of haptoglobin at D2 and cytokines at D1, percentage of granulocytes (CD172a+SWC8+) and macrophages (CD172a+CD203a+) at D1 and D7, post-vaccinal responses (IFNγ-SC and neutralizing antibody (NA)) at D0 and number of IFNγ-SC and NA at D21. Analysis performed with data obtained in H1N2 and V+H1N2 groups.

	Excretion	Haptoglobin	IL-6	TNF-α	IFN-α	Granulocytes D1	Macrophages D1	Granulocytes D7	Macrophages D7	IFNγ-SC D0	H1_hu_N2-Specific NA D0	IFNγ-SC D21	H1_hu_N2-Specific NA D21	H1_hu_N2_Δ146__-147_-Specific NA D21
clinical score	0.37	0.41	0.29	0.23	0.52	0.52	−0.41	0.52	−0.29	−0.41	−0.31	−0.06	−0.24	−0.26
excretion		0.87	0.74	0.25	0.73	0.28	−0.41	0.15	−0.72	−0.62	−0.93	−0.49	−0.31	−0.75
haptoglobin			0.92	0.17	0.52	0.16	−0.31	0.14	−0.70	−0.73	−0.83	−0.52	−0.65	−0.88
IL-6				0.13	0.41	0.20	−0.37	0.22	−0.76	−0.68	−0.82	−0.50	−0.61	−0.86
TNF-α					0.72	0.32	−0.37	0.67	−0.18	0.07	−0.16	−0.46	−0.17	−0.43
IFN-α						0.46	−0.56	0.61	−0.53	−0.35	−0.63	−0.44	−0.09	−0.52
granulocytes D1							−0.94	0.49	−0.39	0.15	−0.36	0.08	−0.12	−0.19
macrophages D1								−0.52	0.47	−0.05	0.48	−0.04	0.18	0.36
granulocytes D7									−0.37	−0.29	−0.28	−0.32	0.07	−0.21
macrophages D7										0.58	0.85	0.72	0.35	0.49
IFNγ-SC D0											0.69	0.47	0.17	0.48
H1_hu_N2-specific NA D0												0.52	0.22	0.70
IFNγ-SC D21													0.35	0.44
H1_hu_N2-specific NA D21														0.65

Grey cells indicate that the correlation is significant at *p* ≤ 0.05.

**Table 7:** Correlation analysis between parameters obtained in vaccinated piglets infected with the variant 212/13 strain. Spearman’s correlation coefficients between clinical score at D1, duration of viral excretion, concentrations of haptoglobin at D2 and cytokines at D1, percentage of granulocytes (CD172a+SWC8+) and macrophages (CD172a+CD203a+) at D1 and D7, post-vaccinal responses (IFNγ-SC and neutralizing antibody (NA)) at D0 and number of IFNγ-SC and NA at D21. Analysis performed with data obtained in H1N2var and V+H1N2var groups.

	Excretion	Haptoglobin	IL-6	TNF-α	IFN-α	Granulocytes D1	Macrophages D1	Granulocytes D7	Macrophages D7	IFNγ-SC D0	H1_hu_N2-Specific NA D0	IFNγ-SC D21	H1_hu_N2-Specific NA D21	H1_hu_N2_Δ146__-147_-Specific NA D21
clinical score	0.71	0.19	0.61	0.42	0.23	0.39	−0.75	0.77	−0.84	−0.75	−0.73	−0.68	−0.73	−0.63
excretion		0.50	0.88	0.46	0.31	0.54	−0.75	0.81	−0.82	−0.75	−0.81	−0.75	−0.79	−0.83
haptoglobin			0.41	0.07	0.18	0.27	−0.12	0.54	−0.02	−0.46	−0.60	−0.73	−0.53	−0.55
IL-6				0.57	0.48	0.16	−0.40	0.63	−0.87	−0.81	−0.77	−0.55	−0.76	−0.81
TNF-α					0.91	0.10	−0.36	0.27	−0.51	−0.23	−0.28	−0.26	−0.29	−0.15
IFN-α						0.13	−0.24	0.22	−0.30	−0.17	−0.17	−0.15	−0.18	0.01
granulocytes D1							−0.90	0.68	−0.31	−0.14	−0.24	−0.45	−0.30	−0.26
macrophages D1								−1.00	0.64	0.55	0.63	0.69	0.48	0.47
granulocytes D7									−0.70	−0.74	−0.70	−0.72	−0.63	−0.67
macrophages D7										0.87	0.79	0.52	0.72	0.71
IFNγ-SC D0											0.93	0.71	0.76	0.81
H1_hu_N2-specific NA D0												0.90	0.84	0.81
IFNγ-SC D21													0.72	0.70
H1_hu_N2-specific NA D21														0.76

Grey cells indicate that the correlation is significant at *p* ≤ 0.05.

## Supplementary Materials

The following are available online at https://www.mdpi.com/1999-4915/12/10/1155/s1, Figure S1: Comparison of amino acid sequences of hemagglutinin subunit HA1 of influenza A/H1N2 viruses used in this study.
