# Supplementary material for: Evaluation of the Pathogenicity and the Escape from Vaccine Protection of a New Antigenic Variant Derived from the European Human-Like Reassortant Swine H1N2 Influenza Virus
Source: Viruses. 2020 Oct 12;12(10):1155. doi: 10.3390/v12101155 (PMC7599989; doi:10.3390/v12101155)
Supplement: Supplementary file 1 [file viruses-12-01155-s001.pdf]

```
                                           *        20         *        40         *        60         *        80         *
AF085413|A/Sw/Scotland/410440/1994 : MKEKLLVLLCALSATDADTICIGYHANNSTDTVDTVLEKNVTVTHSVNLLEDSHNGKLCRLKGIAPLQLGKCSIAGWILGNPECELLFSKKS
GQ161104|A/sw/Bakum/1832/2000      : MRAKLLILLCALSATDADTICIGYHANNSTDTVDTVLEKNVTVTHSVNLLEDSHNGKLCRLKGIAPLQLGKCSIAGWILGNPECESLFSKKS
AM777812|A/sw/CA/0214/2006         : MKAKLIVLLCALSATDADTICIGYHANNSTDTVDTVLEKNVTVTHSVNLLEDSHNGKLCRLKGIAPLQLGKCSIAGWILGNPECEPLFSRKS
AM503902|A/sw/CA/0113/2006         : MKAKLLILLCALSATDADTICIGYHANNSTDTVDTVLEKNVAVTHSVNLLEDSNNGKLCRLKGIPPLHLGRCSIAGWILGNPECESLFSKKS
KR699790|A/sw/Fr/IV_0415/2011      : MKAKLLILLCTLSATDADTICIGYHANNSTETVDTVLEKNVTVTHSVNLLEDSHNGKLCRLKGIPPLQLGRCSIAGWILGNPECESLFSKKS
KJ128323|A/sw/Fr/22-130212/2013    : MKAKLLILLCTLSATDADTICIGYHANNSTETVDTVLEKNVAVTHSVNLLEDSHNGKLCRLKGIPPLQLGRCSIAGWILGNPECESLFSKKS

                                                                                     Del 146
                                         100         *       120         *       140  -147 130 loop   160         *       180
AF085413|A/Sw/Scotland/410440/1994 : WSYIAETPNSENGICYPGYFSDYEELREQLSSVSSFERFEIFPKESSWPKHSVNKGVTKSCSHKGKSSFYRNLLWLTEKNGSYPNLSKSYVN
GQ161104|A/sw/Bakum/1832/2000      : WSYIAETPNSENGICYPGYFSDYEELREQLNSVSSFERFEIFPKEXSWPKHSVNKGVTAACSHEGKSSFYRNLLWLTERNGSYPNLSKSYVN
AM777812|A/sw/CA/0214/2006         : WSYIAETPNSEYGVCYPGYFSDYEELREQLSSVSSFERFEIFPKESSWPKHNVSKGVTASCSHKGKSSFYRNLLWLTEKDGSYPNLSKPYVN
AM503902|A/sw/CA/0113/2006         : WSYIAETPNPENGICYPGYFSDYEELREQLSSVSSFERFEIFPKESSWPKHSINKGVTASCSHKGKRSFYKNLLWLTEKNGSYPNLSKSYVN
KR699790|A/sw/Fr/IV_0415/2011      : WSYIAETSNXEYGICYPGYFSDYEELREQLSSVSSIERFEIFPKESSWPKHSINKGVTASCSHKGKSSFYRNLLWLTETNGSYPNLSKSYVN
KJ128323|A/sw/Fr/22-130212/2013    : WSYIAETSNPEYGICYPGYFSDYEELREQLSSVSSFERFEIFPKESSWPKHSI--GATASCSHKGESSFYRNLLWLTEKNGSYPKLSMSYVN

                                                     190 helix                     220 loop
                                         *       200         220         *             *       260         *
AF085413|A/Sw/Scotland/410440/1994 : NKEKEVLVLWGVHHPSNIENQKTIYRKETAYVSVVSSHYNRRFTPEITRRPKVRDQEGRINYYWTLLEPGDTIIFEANGNLIAPWYRFALSR
GQ161104|A/sw/Bakum/1832/2000      : NREEEVLVLWGVHHPSNIEDQRAIYRKETAYVSVVSSHYNRRFTPEIAKRPKVRGQEGRINYYWTLLEPRDTIIFEANGNLIAPWYAFALSR
AM777812|A/sw/CA/0214/2006         : NKEKEVLILWGIHHPSNIEDQRAIYRKETAYVSVVSSHYNRRFTPEIAKRPKIRNQEGRINYYWTLLEPGDTIIFEANGNLIAPWYAFALNR
AM503902|A/sw/CA/0113/2006         : DKGKEVLVLWGVHHPSNIEDQRAIYRKENAYVSVVASHYNRRFTPEIARRPKIRDQEGRINYYWTLLEPSDTIIFEANGNLIAPWYAFALSR
KR699790|A/sw/Fr/IV_0415/2011      : DKGKEVLVLWGVHHPSNIEDQRAIYRKETAYVSVVASHYNRRFTPEVARRPKIRDQEGRINYYWTLLGPRDTIIFEANGNLIAPWYAFALSR
KJ128323|A/sw/Fr/22-130212/2013    : DKEKEVLVLWGVHHPSNIEDQRAIYRKETAYVSVMASHYNRRFTPEIAKRPKIRNQEGRINYYWTLLGPRDTIMFEANGNLIAPWYAFALSR

                                       280         *       300         *       320         *       340
AF085413|A/Sw/Scotland/410440/1994 : GFGSGIIISNASMDECDAKCQTPQGAINSSLPFQNVHPVTIGECPKYVRSKKLRMVTGLXNIPSIQSR
GQ161104|A/sw/Bakum/1832/2000      : GFESGIIVSNASMDECDTKCQTPRGAINSSLPFQNVHPVTIGECPKYVRSTKLRMVTGLRNIPSIQSR
AM777812|A/sw/CA/0214/2006         : GFESGIIITNASMDECDTKCQTPQGAINSSLPFQNVHPVTIGECPKYVRSTKLRMVTGLRNIPSIQSR
AM503902|A/sw/CA/0113/2006         : GFRSGIIVSNASMDECDAKCQTPQGAINSSLPFQNVHPVTIGECPKYVRSTKLRMVTGLR
KR699790|A/sw/Fr/IV_0415/2011      : GFKSGIIISNASMGECDTKCQTPQGAINSSLPFQNVHPVTIGECPKYVRSTKLRMVTGLRNIPSIQSR
KJ128323|A/sw/Fr/22-130212/2013    : GFKSGIIISNASIGECDAKCQTPQGAINSSLPFQNVHPVTIGECPKYVRSTKLRMATGLRNIPSIQSR
```

Figure S1 : Comparison of amino acid sequences of hemagglutinin subunit HA1 of influenza A/H1N2 viruses used in this study.

Alignment of amino acid sequences of predicted HA1 proteins numbered from the first methionine (including peptide signal). Amino acid changes from the reference A/Sw/Scotland/410440/1994(H1N2) are indicated in red. Antigenic sites are in bold; RBS are highlighted in yellow according to Sriwilaijaroen and Suzuki 2012 (Sriwilaijaroen N, Suzuki Y. Molecular basis of the structure and function of H1 hemagglutinin of influenza virus. *Proc Jpn Acad Ser B Phys Biol Sci*. **2012**;88(6):226-49. doi: 10.2183/pjab.88.226.)
